# A Network Pharmacology Approach to Uncover the Potential Mechanism of Yinchensini Decoction

**DOI:** 10.1155/2018/2178610

**Published:** 2018-12-20

**Authors:** Guoming Chen, Chuyao Huang, Yunyun Liu, Tengyu Chen, Ruilan Huang, Miaozhen Liang, Jie Zhang, Hua Xu

**Affiliations:** ^1^Guangzhou University of Chinese Medicine, Guangzhou, China; ^2^Department of Paediatrics, First Affiliated Hospital of Guangzhou University of Chinese Medicine, Guangzhou, China

## Abstract

**Objective:**

To predict and explore the potential mechanism of Yinchensini decoction (YCSND) based on systemic pharmacology.

**Method:**

TCMSP database was searched for the active constituents and related target proteins of YCSND. Cytoscape 3.5.1 was used to construct the active ingredient-target interaction of YCSND and network topology analysis, with STRING online database for protein-protein interaction (PPI) network construction and analysis; and collection from the UniProt database of target protein gene name, with the DAVID database for the gene ontology (GO) functional analysis, KEGG pathway enrichment analysis mechanism and targets of YCSND.

**Results:**

The results indicate the core compounds of YCSND, namely, kaempferol, 7-Methoxy-2-methyl isoflavone, and formononetin. And its core targets are prostaglandin G/H synthase 2, estrogen receptor, Calmodulin, heat shock protein HSP 90, etc. PPI network analysis shows that the key components of the active ingredients of YCSND are JUN, TP53, MARK1, RELA, MYC, and so on. The results of the GO analysis demonstrate that extracellular space, cytosol, and plasma membrane are the main cellular components of YCSND. Its molecular functions are mainly acting on enzyme binding, protein heterodimerization activity, and drug binding. The biological process of YCSND is focused on response to drug, positive regulation of transcription from RNA polymerase II promoter, the response to ethanol, etc. KEGG results suggest that the pathways, including pathways in cancer, hepatitis B, and pancreatic cancer, play a key role in YCSND.

**Conclusion:**

YCSND exerts its drug effect through various signaling pathways and acts on kinds of targets. By system pharmacology, the potential role of drugs and the mechanism of action can be well predicted.

## 1. Introduction

Yinchensini decoction (YCSND) is a classical traditional Chinese medicine (TCM) prescription which has originated and been in usage since Song dynasty. YCSND is composed of four Chinese medicinal herbs, namely, Artemisiae scopariae herba (*Yinchen*), Radix aconiti Carmichael (*Fuzi*), Rhizoma zingiberis (*Ganjiang*), and Liquorice (*Gancao*). Pharmacologic studies have shown that Yinchen and Gancao have an effect on protecting liver and opposing hepatitis virus and also have the cholagogue action and anti-inflammatory effect. Then, Fuzi has effects in enhancing immunity, combating inflammation, and easing pain, and Ganjiang plays a part in protecting the liver, benefitting the bile, improving blood circulation, and stopping vomiting [1, 2]. Moreover, a preexisting experimental study has shown that YCSND has been extensively put into clinical use for the treatment of Yin jaundice and liver diseases [3]. Chinese herbal compound prescription is composed of many different compounds with various structures and functions, and it is unscientific that a specific effective chemical compound contains its entire medicinal value. Many components act on its mechanism through multiple targets instead of a specific target. As far as known, experimental studies of YCSND have been reported, but the specific mechanism is not fully clear. Thus, to illustrate its mechanism more systematically and comprehensively, this research intends to analyze and expound the potential molecular mechanism of YCSND based on system pharmacology. As an emerging discipline, systems pharmacology includes many disciplines such as systems biology, pharmacology, computational biology, and network analysis, which to a great degree break the traditional framework (drug-target-disease)[4]. Constructing a multilevel network (disease-phenotype-gene-drug) and exploring the correlation between drugs and disease from the perspective of the whole, which has the characteristics of wholeness and systematicness, correspond with the principle of a holistic view and dialectical treatment of TCM.

Therefore, based on the characteristics and methods of system pharmacology, the analysis of the existing data and the collation of target points and their chemical molecules are carried out. Through analyzing the potential interaction between the various target points, the network of target points is constructed. Then the analysis of associated pathological pathways and the summary of the potential mechanism of YCSND are achieved.

## 2. Materials and Methods

### 2.1. Constructing Database of Candidate Compounds

In the Traditional Chinese Medicine Systems Pharmacology Database and Analysis Platform (http://lsp.nwu.edu.cn/tcmsp.php,TCMSP), five constituents of Yinchensini decoction are retrieved. A total of 106 compounds are achieved. Each candidate's druggability was analyzed according to its oral bioavailability (OB) and drug-likeness (DL) indices recommended by TCMSP. OB refers to the degree and speed of absorbing drugs into the circulatory system, which is an important indicator to evaluate the intrinsic quality of drugs objectively. The higher the OB of the compound is, the more likely the compound is to be developed for clinical application. DL is the sum of the pharmacokinetic properties and safety, which comes from the interactions of physicochemical properties and structural factors, including solubility, permeability, and stability. It can be used to optimize compounds, analyze the results of drug activity, predict in vivo pharmacokinetics, direct structure modifications, etc. As TCMSP recommends, the molecules with OB≥30% and DL≥0.18 were considered to exhibit relatively better pharmacologically and were screened out as candidate compounds for further analysis.

### 2.2. Constructing the Network of Compound-Target

To comprehensively understand the molecular mechanisms, the compound-target networks were constructed using Cytoscape visualization software 3.5.1. All the candidate compounds were retrieved in TSMSP to obtain associated targets. Then, compounds and targets were inputted into the software and compound-target interaction network was carried out. In the process of constructing the network, the layout algorithm (attribute circle layout) was applied. Users can set the geometric position of every node and display visually network topology using color, graphics, symbols, making reasonable arrangements of every node and creating a clear visual effect. Degree and betweenness centrality are two important parameters of the topology structure, which were used to evaluate the essentiality of each target and compound. Therefore, targets and compounds that play a key role in the mechanisms of Yinchensini decoction were revealed and analyzed.

### 2.3. Conducting PPI Network

Since the chances of proteins achieving assigned functions individually are small, which means proteins involved in the biochemical process in the same cell tend to form macromolecular complexes through the interactions to complete biological functions; so, the exploration of protein interactions and the interaction network is the viral procedure of understanding cellular organization, bioprocess, and functions. In order to better understand protein interactions systematically, associated targets were input to STRING 10.5 (Search Tool for the Retrieval of Interacting Genes/Proteins) to obtain relevant information of protein interaction. STRING is a commonly used system for retrieval or prediction of protein-protein interaction, known interaction, predicted interaction, and others included. The network nodes represent proteins, and edges represent protein-protein associations. Its results are derived from experimental data, literature mining, databases, and bioinformatics projections. The scoring mechanism of the system itself can score the results from different paths, and the higher the score is, the higher the confidence of the protein action information is. To ensure high confidence information, the minimum score was set to the highest confidence as 0.9. Also, disconnected proteins in the network were excluded. Last, PPT network was exported and, according to it, statistics of protein interactions were carried out.

### 2.4. Gene Ontology (GO) Functional Enrichment Analysis

Gene Ontology (GO) Consortium database is established by Gene Ontology Consortium, which can describe and limit the functions of genes, and is applicable to all species. The object of this study is a group of genes, and if they are directly annotated, the number of functional nodes obtained is large and overlapping, which results in redundancy. Therefore, the data were analyzed by functional enrichment. The method can effectively identify biological processes related to biological phenomena and is useful for obtaining more meaningful gene functional information. GO enrichment analysis was performed using the functional annotation tool of DAVID (Database for Annotation, Visualization, and Integrated Discovery). Before the enrichment, protein names of all targets were converted into corresponding gene names in the website of UniProt, and then gene names were imported into DAVID to acquire GO enrichment analysis.

### 2.5. KEGG Pathway Enrichment Analysis

KEGG ((Kyoto Encyclopedia of Genes and Genomes) is a database developed by University of Tokyo and Kyoto University, Japan, which provides query path databases. The identification codes converted from UniProt were imported DAVID access database. Next, the pathway enrichment of all protein genes was conducted, and the KEGG pathway annotations were analyzed, to explore biological pathways which related proteins were involved in.

## 3. Results

### 3.1. Identification of the Active Compounds in YCSND

Using the TCMSP database, 546 compounds were retrieved: 53 in Yinchen, 65 in Fuzi, 148 in Ganjiang, and 280 in Gancao. With the criteria of OB≥30% and DL≥0.18, 131 chemical ingredients were screened out: 13 in Yinchen, 21 in Fuzi, 5 in Ganjiang, and 92 in Gancao. As shown in Table 1, actually 126 chemical constituents were accepted in our study after taking out the duplicated parts.

### 3.2. Constructing the Network of Compound-Target

**Figure 1:**
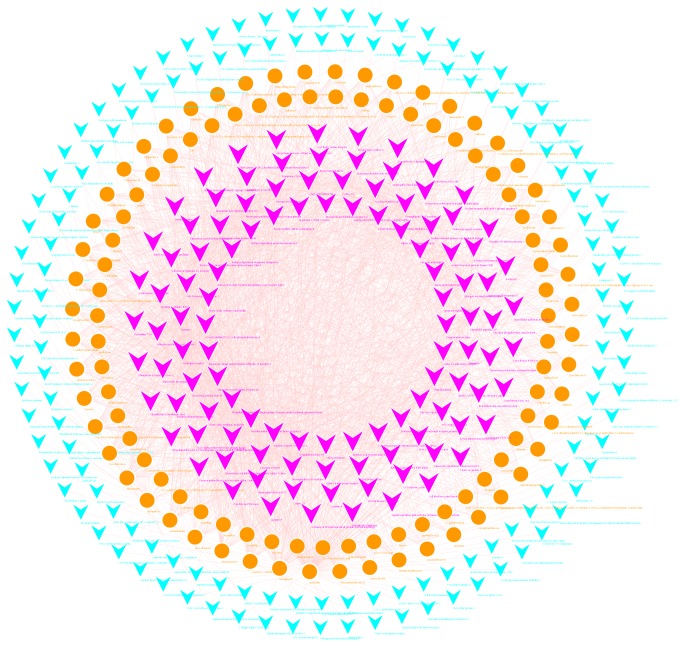
The compound-target network of YCSND.

After importing data into Cytoscape 3.5.1, compound-target network (Figure 1) was constructed. In the network, the orange node (107) represents the YCSND active compound; the blue (138) and purple (112) nodes represent the target protein; the network contains 357 nodes and 1986 edges in total; the degree of a node indicates the number of routes in which the network is connected to the node. The outer blue node is the target which degree is 1; the inside purple node is of the degree more than 2 of the target (communicate orange node). Results of the network topology analysis are as follows: network density (0.031), network heterogeneity (1.603), and shortest paths (127092, 100%). The average degree of nodes is 11.12605, and there are 112 nodes larger than the average degree. The average betweenness centrality of nodes is 0.00529, and there are 52 nodes larger than the average betweenness centrality. The key core nodes (compound or target) are screened based on the topological properties of degree and betweenness centrality of network nodes, as shown in Table 2. It is suggested that the connections between key compounds and target nodes play a pivotal role in the network.

### 3.3. Analysis of the Targets in the PPI Network

**Figure 2:**
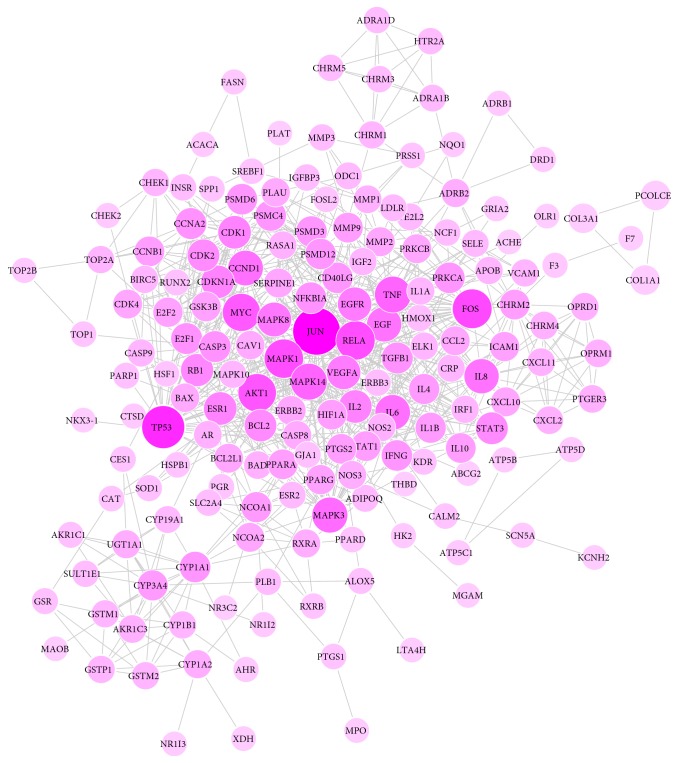
The PPI network of YCSND (the larger the node, the deeper the color, representing the greater the degree of the node).

PPI network is conducted to better analyze and understand the mechanisms of YCSND based on the study of protein-protein interactions by using STRING software. A total of 856 interrelations, as well as 179 related targets, are obtained in PPI network after setting the confidence level greater than 0.9 and rejecting the target protein independent of the network. The importance prioritization of key proteins is analyzed according to the degree of the node exported from STRING database. Among them, the JUN value (degree=54) is much higher than that of other protein nodes, which indicates that this protein might play a role of bridge to connect other nodes in PPI network (Figure 2). The PPI network combined scores were showed in supplementary information file (available here).

### 3.4. Gene Ontology (GO) Functional Enrichment Analysis

GO annotation and enrichment of YCSND target protein genes in three aspects of cell composition (CC), molecular function (MF), and biological process (BP) were carried out through the DAVID database. The enrichment results showed that there were 79 enrichment results in the related items of cell composition, involving extracellular space, cytosol, plasma membrane, and other cell components; 161 enrichment results are related to molecular function, which includes enzyme binding, protein heterodimerization activity, and drug binding; 748 enrichment processes are related to the biological processes which cover the response to drug, positive regulation of transcription from RNA polymerase II promoter, response to ethanol, etc. Each p value of enrichment results was calculated (corrected by using the Bonferroni method, p values < 0.01 were considered to be significantly enriched), ranking p values according to the order from small to large. The top 10 enrichment results are displayed, and details are shown in Tables 3, 4, and 5.

### 3.5. KEGG Pathway Enrichment Analysis

The related pathway of YCSND was obtained by KEGG pathway enrichment analysis through the DAVID database. 135 pathways are enriched, and each p value of enrichment results was calculated (corrected by using the Bonferroni method, p values < 0.01 were considered to be significantly enriched). After sorting the p values, the top 10 are analyzed (Table 6).

## 4. Discussion

Yinchensini decoction, first recorded in Song dynasty, which can warm Yang to improve jaundice as well as excrete excess water, is used for hepatitis, jaundice, biliary atresia, liver cancer etc. Based on the theory of traditional Chinese medicine, the main indications of Yinchensini decoction includes Yin jaundice, cold extremities, sweating, and drop in blood pressure. As a new subject, network pharmacology can build a component-target network, combine with the proven literature to demonstrate the mechanism of prescription on diseases, and predict the possible mechanism. As the Yinchensini decoction implements its efficacy through multiple targets and multiple approaches, we used network pharmacology to break the limitation of single pharmacology study and explored the mechanism of Yinchensini decoction for its indications more comprehensively. Through algorithm statistics for GO enrichment and KEGG pathway enrichment, we found that the important components of Yinchensini decoction, such as quercetin and kaempferol, may act on key targets, including JUN, RELA, and IL-6, ultimately improving jaundice, inhibiting inflammatory factors, promoting cell proliferation, and regulating RNA polymerase II transcription factor activity via TNF signaling pathway, bladder cancer, and other pathways.

In vitro, quercetin can not only inhibit the proliferation and apoptosis of many tumor cells through multiple signaling pathways, such as the Wnt signaling pathway (cholangiocarcinoma) and the JNK signaling pathway, but enhance the sensitivity of other anticancer drugs and reverse the drug resistance of tumor cells [5]. What is more, Quercetin has all kinds of pharmacological activities such as antioxidant and hepatoprotective effects [6]. It is reported that quercetin can attenuate oxidative stress in alcohol-induced liver disease via heme oxygenase-1 restoration, decreased lipid oxidation, and diminished ROS generation [7].

Prostaglandin G/H synthase 2(PTGS2/COX-2) is closely tied with cancer [8]. Meanwhile, COX-2, as an inflammatory factor, can cause inflammation and oxidative stress injury. COX-2, involved in prostaglandin synthesis, can be detected in several liver pathologies [9]. It is known that liver ischemia-reperfusion injury is common in liver transplantation, shock or acute hemorrhage, with cold limbs and hypotension. Several reports supported that hepatocyte-specific constitutive expression of COX-2 plays a protective role in liver ischemia-reperfusion injury by diminished proinflammatory cytokines (i.e., IL-1*β*, IL-6, and TNF-*α*), increased antiapoptosis (i.e., BAX/BCL-2 radio), and activated AKT and AMPK [10–12]. Previous studies have suggested that calmodulin is relevant to high-grade serous ovarian cancer [13]. As a high frequency of complications in chronic cholestasis, hypogonadism was observed on the ovary of adult cycling rats with chronic obstructive jaundice, which lead to marked stromal fibrosis and diminished expression of estrogen receptors [14]. Kaempferol and some glycosides of kaempferol have a wide range of pharmacological activities, such as antioxidant, anti-inflammatory, antimicrobial, anticancer, antidiabetic, antiosteoporotic, anxiolytic, analgesic, and antiallergic activities as numerous preclinical studies have shown [15]. Kaempferol is one of the active fractions in Glycosmis pentaphylla (Retz.) DC, which is traditionally used for the treatment of rheumatism, anemia, jaundice, bronchitis, etc. [16].

PPI network analysis shows that score and confidence level of JUN, TP53, FOS, MAPK1, RELA, MYC, MAPK14, MAPK3, EGF, IL6, IL8, and MAPK8 were significantly higher than others. The coding genes of JUN and FOS target protein belong to the immediate early genes of the protooncogenes, which can rapidly be expressed under the stimulation of external factors. The expression products, FOS and JUN, form heterodimer FOS: JUN or homodimer JUN: JUN in a series of the modification process, then combine with the binding sites of activated protein 1(AP -1), and at last have an effect on the expression of target genes [17]. The expression and activity regulation of c-jun is regulated by various protein kinases which play the role of active sites of signaling pathways. C-jun is also involved in the process of tumor cell growth regulation in various growth factors, cytokines and extracellular stimuli [18].

At present, many experimental studies have confirmed that high c-jun expression is highly correlated with the occurrence and prognosis of various malignant tumors [19]. For example, Yang yuewu [20] found that the expression of c-jun in hepatocellular carcinoma (HCC) correlates with HBsAg, AFP, tumor diameter, tumor capsule, tumor vascular invasion and so on, suggesting the c-jun may play an important role in the occurrence and development of liver cancer. RELA is also known as nuclear factor kappa B, a nucleoprotein factor with multidirectional transcriptional regulation effect, which widely exists in various cells of mammals. RELA can activate a variety of related gene transcription and participate in the cell carcinogenesis so that both are confirmedly related to cell growth and apoptosis [21]. A study found that the positive expression rate of the RELA in the tissue of HCC is significantly higher than that of liver tissue adjacent to carcinoma, suggesting that the RELA is closely related to the occurrence of HCC. At the same time, the experiment points out that the expression of the RELA is associated with the malignant degree of tissue of HCC. If cancer tissue becomes worse on differentiation, the expression of RELA will be higher. It can speculate that the RELA could control the transcription of the downstream antiapoptotic gene, thus inhibiting cell apoptosis of liver cancer and causing the proliferation ability of hepatoma carcinoma cell to be strengthened [22].

EGF is an effective mitogenic factor that can stimulate cell division and proliferation in multiple tissues and promote infiltration and metastasis of tumor cells. EGFR is a kind of cell membrane protein kinase receptor which plays a key role in maintaining cell growth and proliferation.

Its excessive activation can spur proliferation and inhibit apoptosis of malignant tumor cells and also can promote tumor metastasis and angiogenesis [23]. The study shows that EGF and EGFR can promote DNA synthesis of HCC by means of the ion channel, signal conduction, and gene expression, thus promoting the occurrence and development of HCC [24]. Jaundice is one of the main symptoms of HCC and YCSND is used to treat Yin jaundice which is a pattern of jaundice in TCM in the clinic. It is speculated that the YCSND can treat jaundice by inhibiting the expression of c-jun, RELA, EGF, and EGFR. In addition, RELA plays an important role in the body's immune and inflammatory response and apoptosis regulation and its excessive activation can cause many kinds of pathophysiological reaction. Abdominal pain, nausea, vomiting, fever, and jaundice can be often seen in acute pancreatitis (AP). And the excessive activation of RELA can raise gene expression related to a variety of inflammatory responses in the occurrence and development process of acute pancreatitis, causing large numbers of cytokines and inflammatory mediators being involved in the inflammatory process of AP [25]. Studies have shown that inflammation of the pancreas can be improved effectively by inhibiting activation of RELA, which can reduce expression of TNF alpha mRNA [26].

IL-6 is one of the most biologically active cytokines and has many biological functions. In recent years, numerous experiments have confirmed that the abnormal expression of IL-6 and its receptor is associated with the pathogenesis of tumor and is related with the diagnosis, prognosis, and treatment of tumor [27]. It is reported that the concentration of IL-6 is significantly higher than normal levels in the patients with bile duct carcinoma (BDC), speculating that IL6 has the diagnostic significance of BDC [28]. At the same time, IL6 has an antitumor effect, which can directly or indirectly enhance the tumor-cytotoxic effect of the natural killer cell and cytotoxic lymphocytes [29]. Jaundice is the primary symptom of BDC and YCSND is used to treat Yin jaundice clinically. Thus YCSND is speculated to treat BDC by enhancing the antitumor effect of IL6. MAPK signaling pathway is one of the important signaling pathways of organisms, which is involved in the physiological processes of the cell, such as inflammatory reaction, cell growth, cell differentiation, cell proliferation, and cell survival. MAPK1 is also called ERK2. c-fos is its downstream target gene, closely related to the tumor malignant transformation and proliferation. In addition, MAPK14 belongs to a stress-induced type of MAPK family and the activated MAPK14 highly link to the expression of the downstream gene, c-myc, and induce transposition of BAX as well as enhance the expression of TNF alpha and induce cell physiological dysfunction and apoptosis by activating the hippocampus [30]. Many Chinese herbal medicines and their derivatives can prevent liver cancer by inhibiting the MAPK signaling pathway, such as Phyllanthus amarus, Benzyl sulforaphane, and Schizocarps plantaginea [31–33]. So it can be speculated that YCSND can treat jaundice caused by HCC or AP by inhibiting MAPK signaling pathway which can inhibit inflammation and tumor proliferation.

As the purpose of this paper is to explore how YCSND plays its therapeutic role through its effective components acting on multiple targets and multiple pathways, KEGG were used to enrich pathways. And based on a large number of reported literatures, each link of the signal pathways is depicted in the KEGG database. Therefore, we can analyze the cellular components, biochemical processes, and molecular functions from the enriched pathways to corroborate the GO enriched results.

Hepatitis B is one of the significantly enriched pathways, which composes of many signal pathways and involves in complex biological processes. Double-stranded relaxed circular DNA (RC-DNA) is the main genetic material of hepatitis B virus. After entering the hepatocyte nucleus, RC-DNA is transformed into cccDNA. Then all viral RNAs including the pregenomic RNA (pgRNA) start transcribing through cccDNA, and HBV core and polymerase II are translated [34]. Ren JH [35] found that SIRT3 restricts the transcription of HBV by decreased host RNA polymerase II and transcription factor binding. Thus, YCSND may inhibit reverse transcription of HBV by regulating RNA polymerase II and transcription factors, which reduce the inflammatory response in HBV patients.

It is well known that many proteins are embedded in the surface of cell membrane and endoplasmic reticulum, which mediate biochemical reactions in the body and thus guarantee normal life activities. TNF is mainly produced by activatory mononuclear macrophages and is an important inflammatory factor. Activated TNF binds to its receptors (TNFR1, TNFR2) in the cell membrane resulting in the activation of many genes and initiating NF-kappa B pathway and the MAPK pathway [36]. TNF signaling pathway can mediate the inflammatory immune response together with the positive regulation of mRNA expression of transcription factors (c-fos, c-jun) and the level of inflammatory cytokines etc. [37]. There is a research showing that a large amount of TNF alpha which can be involved in inducing the expression of IL-1, IL-6, IL-8, and its own genes can be released in process of AP, resulting in a large release of cytokines and inflammatory mediators and causing the necrosis of pancreatic tissue [38]. Thus TNF signaling pathway is involved in the occurrence and development of AP as an important proinflammatory cytokine. Jaundice is one of the main symptoms of AP and YCSND is used to treat Yin jaundice. So it can be speculated that YCSND can treat jaundice by inhibiting the expression of TNF signaling pathway. Subsequently, the inflammation of AP is reduced because the generation of inflammatory mediators and cytokines is reduced.

## Figures and Tables

**Table 1:** Information for 126 chemical ingredients of YCSD.

**Mol ID**	**Molecule Name**	**OB**%	**DL**	**Source**
MOL004609	Areapillin	48.96	0.41	Yinchen
MOL005573	Genkwanin	37.13	0.24	
MOL007274	Skrofulein	30.35	0.3	
MOL008039	Isoarcapillin	57.4	0.41	
MOL008040	Eupalitin	46.11	0.33	
MOL008041	Eupatolitin	42.55	0.37	
MOL008043	Capillarisin	57.56	0.31	
MOL008045	4'-Methylcapillarisin	72.18	0.35	
MOL008046	Demethoxycapillarisin	52.33	0.25	
MOL008047	Artepillin A	68.32	0.24	
MOL002211	11,14-eicosadienoic acid	39.99	0.2	Fuzi
MOL002388	Delphin_qt	57.76	0.28	
MOL002392	Deltoin	46.69	0.37	
MOL002393	Demethyldelavaine A	34.52	0.18	
MOL002394	Demethyldelavaine B	34.52	0.18	
MOL002395	Deoxyandrographolide	56.3	0.31	
MOL002397	Karakoline	51.73	0.73	
MOL002398	Karanjin	69.56	0.34	
MOL002401	Neokadsuranic acid B	43.1	0.85	
MOL002406	2,7-Dideacetyl-2,7-dibenzoyl-taxayunnanine F	39.43	0.38	
MOL002410	Benzoylnapelline	34.06	0.53	
MOL002415	6-Demethyldesoline	51.87	0.66	
MOL002416	deoxyaconitine	30.96	0.24	
MOL002419	(R)-Norcoclaurine	82.54	0.21	
MOL002421	Ignavine	84.08	0.25	
MOL002422	Isotalatizidine	50.82	0.73	
MOL002423	Jesaconitine	33.41	0.19	
MOL002433	(3R,8S,9R,10R,13R,14S,17R)-3-hydroxy-4,4,9,13,14-pentamethyl-17-[(E,2R)-6-methyl-7-[(2R,3R,4S,5S,6R)-3,4,5-trihydroxy-6-[[(2R,3R,4S,5S,6R)-3,4,5-trihydroxy-6-(hydroxymethyl)oxan-2-yl]oxymethyl]oxan-2-yl]oxyhept-5-en-2-yl]-1,2,3,7,8,10,12,15,16,17-decahydr	41.52	0.22	
MOL002434	Carnosifloside I_qt	38.16	0.8	
MOL000538	Hypaconitine	31.39	0.26	
MOL002464	1-Monolinolein	37.18	0.3	
MOL002501	[(1S)-3-[(E)-but-2-enyl]-2-methyl-4-oxo-1-cyclopent-2-enyl] (1R,3R)-3-[(E)-3-methoxy-2-methyl-3-oxoprop-1-enyl]-2,2-dimethylcyclopropane-1-carboxylate	62.52	0.31	Ganjiang
MOL002514	Sexangularetin	62.86	0.3	
MOL004898	(E)-3-[3,4-dihydroxy-5-(3-methylbut-2-enyl)phenyl]-1-(2,4-dihydroxyphenyl)prop-2-en-1-one	46.27	0.31	
MOL004903	Liquiritin	65.69	0.74	
MOL004904	Licopyranocoumarin	80.36	0.65	
MOL004905	3,22-Dihydroxy-11-oxo-delta(12)-oleanene-27-alpha-methoxycarbonyl-29-oic acid	34.32	0.55	
MOL004907	Glyzaglabrin	61.07	0.35	
MOL004908	Glabridin	53.25	0.47	
MOL004910	Glabranin	52.9	0.31	
MOL004911	Glabrene	46.27	0.44	
MOL004912	Glabrone	52.51	0.5	
MOL004913	1,3-dihydroxy-9-methoxy-6-benzofurano[3,2-c]chromenone	48.14	0.43	
MOL004914	1,3-dihydroxy-8,9-dimethoxy-6-benzofurano[3,2-c]chromenone	62.9	0.53	
MOL004915	Eurycarpin A	43.28	0.37	
MOL004917	Glycyroside	37.25	0.79	
MOL004924	(-)-Medicocarpin	40.99	0.95	Gancao
MOL004935	Sigmoidin-B	34.88	0.41	
MOL004941	(2R)-7-hydroxy-2-(4-hydroxyphenyl)chroman-4-one	71.12	0.18	
MOL004945	(2S)-7-hydroxy-2-(4-hydroxyphenyl)-8-(3-methylbut-2-enyl)chroman-4-one	36.57	0.32	
MOL004948	Isoglycyrol	44.7	0.84	
MOL004949	Isolicoflavonol	45.17	0.42	
MOL004957	HMO	38.37	0.21	
MOL004959	1-Methoxyphaseollidin	69.98	0.64	
MOL004961	Quercetin der.	46.45	0.33	
MOL004966	3'-Hydroxy-4'-O-Methylglabridin	43.71	0.57	
MOL000497	licochalcone a	40.79	0.29	
MOL004974	3'-Methoxyglabridin	46.16	0.57	
MOL004978	2-[(3R)-8,8-dimethyl-3,4-dihydro-2H-pyrano[6,5-f]chromen-3-yl]-5-methoxyphenol	36.21	0.52	
MOL004980	Inflacoumarin A	39.71	0.33	
MOL004985	icos-5-enoic acid	30.7	0.2	
MOL004988	Kanzonol F	32.47	0.89	
MOL004989	6-prenylated eriodictyol	39.22	0.41	
MOL004990	7,2',4'-trihydroxy—5-methoxy-3—arylcoumarin	83.71	0.27	
MOL004991	7-Acetoxy-2-methylisoflavone	38.92	0.26	
MOL004993	8-prenylated eriodictyol	53.79	0.4	
MOL004996	gadelaidic acid	30.7	0.2	
MOL000500	Vestitol	74.66	0.21	
MOL005000	Gancaonin G	60.44	0.39	
MOL005001	Gancaonin H	50.1	0.78	
MOL005003	Licoagrocarpin	58.81	0.58	
MOL005007	Glyasperins M	72.67	0.59	
MOL005008	Glycyrrhiza flavonol A	41.28	0.6	
MOL005012	Licoagroisoflavone	57.28	0.49	
MOL005013	18*α*-hydroxyglycyrrhetic acid	41.16	0.71	
MOL005016	Odoratin	49.95	0.3	
MOL005017	Phaseol	78.77	0.58	
MOL005018	Xambioona	54.85	0.87	
MOL001484	Inermine	75.18	0.54	
MOL001792	DFV	32.76	0.18	
MOL000211	Mairin	55.38	0.78	
MOL002311	Glycyrol	90.78	0.67	
MOL000239	Jaranol	50.83	0.29	
MOL002565	Medicarpin	49.22	0.34	
MOL003656	Lupiwighteone	51.64	0.37	
MOL003896	7-Methoxy-2-methyl isoflavone	42.56	0.2	
MOL000392	Formononetin	69.67	0.21	
MOL000417	Calycosin	47.75	0.24	
MOL000422	Kaempferol	41.88	0.24	
MOL004328	Naringenin	59.29	0.21	
MOL004805	(2S)-2-[4-hydroxy-3-(3-methylbut-2-enyl)phenyl]-8,8-dimethyl-2,3-dihydropyrano[2,3-f]chromen-4-one	31.79	0.72	
MOL004806	euchrenone	30.29	0.57	
MOL004808	Glyasperin B	65.22	0.44	
MOL004810	Glyasperin F	75.84	0.54	
MOL004811	Glyasperin C	45.56	0.4	
MOL004814	Isotrifoliol	31.94	0.42	
MOL004815	(E)-1-(2,4-dihydroxyphenyl)-3-(2,2-dimethylchromen-6-yl)prop-2-en-1-one	39.62	0.35	
MOL004820	Kanzonol W	50.48	0.52	
MOL004824	(2S)-6-(2,4-dihydroxyphenyl)-2-(2-hydroxypropan-2-yl)-4-methoxy-2,3-dihydrofuro[3,2-g]chromen-7-one	60.25	0.63	
MOL004827	Semilicoisoflavone B	48.78	0.55	
MOL004828	Glepidotin A	44.72	0.35	
MOL004829	Glepidotin B	64.46	0.34	
MOL004833	Phaseolinisoflavan	32.01	0.45	
MOL004835	Glypallichalcone	61.6	0.19	
MOL004838	8-(6-hydroxy-2-benzofuranyl)-2,2-dimethyl-5-chromenol	58.44	0.38	
MOL004841	Licochalcone B	76.76	0.19	
MOL004848	licochalcone G	49.25	0.32	
MOL004849	3-(2,4-dihydroxyphenyl)-8-(1,1-dimethylprop-2-enyl)-7-hydroxy-5-methoxy-coumarin	59.62	0.43	
MOL004855	Licoricone	63.58	0.47	
MOL004856	Gancaonin A	51.08	0.4	
MOL004857	Gancaonin B	48.79	0.45	
MOL004860	licorice glycoside E	32.89	0.27	
MOL004863	3-(3,4-dihydroxyphenyl)-5,7-dihydroxy-8-(3-methylbut-2-enyl)chromone	66.37	0.41	
MOL004864	5,7-dihydroxy-3-(4-methoxyphenyl)-8-(3-methylbut-2-enyl)chromone	30.49	0.41	
MOL004866	2-(3,4-dihydroxyphenyl)-5,7-dihydroxy-6-(3-methylbut-2-enyl)chromone	44.15	0.41	
MOL004879	Glycyrin	52.61	0.47	
MOL004882	Licocoumarone	33.21	0.36	
MOL004883	Licoisoflavone	41.61	0.42	
MOL004884	Licoisoflavone B	38.93	0.55	
MOL004885	Licoisoflavanone	52.47	0.54	
MOL004891	Shinpterocarpin	80.3	0.73	
MOL005020	Dehydroglyasperin C	53.82	0.37	
MOL000354	Isorhamnetin	49.6	0.31	Yinchen Gancao
MOL000358	Beta-sitosterol	36.91	0.75	Yinchen Ganjiang
MOL000098	Quercetin	46.43	0.28	Yinchen Gancao
MOL000359	Sitosterol	36.91	0.75	Fuzi Ganjiang Gancao

**Table 2:** Important node of YCSND compound-target network.

**Node Name**	**Node Type**	**Degree**	**Betweenness Centrality**
Quercetin	Compound	154	0.51220399
Prostaglandin G/H synthase 2	Target	98	0.09990361
Estrogen receptor	Target	80	0.03095638
Calmodulin	Target	77	0.01919701
Heat shock protein HSP 90	Target	74	0.06433216
Nitric oxide synthase, inducible	Target	74	0.01539794
Androgen receptor	Target	73	0.03326497
Kaempferol	Compound	63	0.10360504
Trypsin-1	Target	63	0.02571407
Cell division protein kinase 2	Target	62	0.00804666
Glycogen synthase kinase-3 beta	Target	61	0.0087541
Peroxisome proliferator-activated receptor gamma	Target	61	0.02602051
Proto-oncogene serine/threonine-protein kinase Pim-1	Target	61	0.00731391
Estrogen receptor beta	Target	60	0.00760087
Coagulation factor Xa	Target	55	0.01878226
Cyclin-A2	Target	53	0.00546001
Nuclear receptor coactivator 2	Target	52	0.05181668
Prostaglandin G/H synthase 1	Target	52	0.04921123
Sodium channel protein type 5 subunit alpha	Target	51	0.02323399
Dipeptidyl peptidase IV	Target	45	0.02031966
7-Methoxy-2-methyl isoflavone	Compound	43	0.0274993
Formononetin	Compound	39	0.0438877
beta-sitosterol	Compound	38	0.06644929
Thrombin	Target	38	0.01452236
Isorhamnetin	Compound	37	0.03169965
Naringenin	Compound	37	0.1201062
Medicarpin	Compound	34	0.02391904
mRNA of PKA Catalytic Subunit C-alpha	Target	34	0.03269624
licochalcone a	Compound	32	0.02913105
2-[(3R)-8,8-dimethyl-3,4-dihydro-2H-pyrano[6,5-f]chromen-3-yl]-5-methoxyphenol	Compound	31	0.00718614
Beta-2 adrenergic receptor	Target	31	0.01440293
DNA topoisomerase II	Target	31	0.00830063
shinpterocarpin	Compound	30	0.01309466
Vestitol	Compound	30	0.00877921
Licoagrocarpin	Compound	29	0.00658159
Retinoic acid receptor RXR-alpha	Target	28	0.01008831
Glypallichalcone	Compound	27	0.00712433
Glyasperins M	Compound	26	0.00616704
Acetylcholinesterase	Target	24	0.00976155
Coagulation factor VII	Target	22	0.00700284
Phosphatidylinositol-4,5-bisphosphate 3-kinase catalytic subunit, gamma isoform	Target	17	0.01365074
Potassium voltage-gated channel subfamily H member 2	Target	16	0.00643202
Nitric-oxide synthase, endothelial	Target	14	0.00565094
Demethoxycapillarisin	Compound	13	0.01972483

**Table 3:** Gene ontology term, cellular component, direct (Top 10).

**GOTERM - CC - DIRECT**	**Count**		**P value**
extracellular space	66	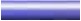	1.2×10^−19^
Cytosol	94	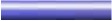	1.3×10^−12^
plasma membrane	103	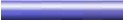	1.3×10^−10^
membrane raft	19	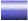	6.0×10^−10^
external side of plasma membrane	18	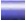	7.2×10^−9^
extracellular exosome	75	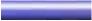	1.3×10^−8^
extracellular region	52	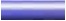	1.5×10^−8^
Caveola	11	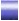	2.0×10^−8^
endoplasmic reticulum membrane	34	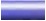	1.2×10^−7^
endoplasmic reticulum	33	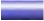	1.5×10^−7^

**Table 4:** Gene ontology term, molecular function, direct (Top 10).

**GOTERM - MF – DIRECT**	**Count**		**P value**
enzyme binding	49	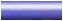	1.4×10^−33^
protein heterodimerization activity	38	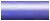	4.9×10^−17^
drug binding	18	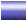	8.2×10^−16^
protein homodimerization activity	45	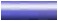	1.6×10^−15^
identical protein binding	42	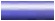	3.9×10^−13^
RNA polymerase II transcription factor activity, ligand-activated sequence-specific DNA binding	12	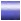	2.7×10^−12^
transcription factor binding	25	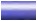	6.7×10^−12^
protein kinase binding	28	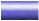	1.4×10^−11^
protein binding	183	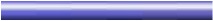	2.0×10^−11^
steroid hormone receptor activity	13	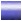	2.8×10^−11^

**Table 5:** Gene ontology term, biological process, direct (Top 10).

**GOTERM - BP - DIRECT**	**Count**		**P value**
response to drug	40	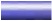	2.2×10^−25^
positive regulation of transcription from RNA polymerase II promoter	58	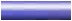	2.0×10^−19^
response to ethanol	22	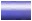	3.6×10^−18^
positive regulation of gene expression	30	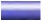	2.6×10^−17^
aging	24	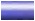	3.8×10^−16^
positive regulation of transcription, DNA-templated	38	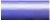	1.5×10^−15^
xenobiotic glucuronidation	9	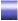	1.9×10^−14^
negative regulation of the fatty acid metabolic process	9	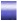	1.9×10^−14^
response to estradiol	18	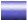	2.2×10^−14^
positive regulation of nitric oxide biosynthetic process	14	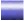	3.2×10^−14^

**Table 6:** KEGG pathway enrichment (Top 10).

**KEGG Pathway**	**Count**		**P value**
Pathways in cancer	60	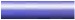	3.7×10^−25^
Hepatitis B	39	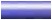	4.0×10^−25^
Pancreatic cancer	28	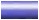	4.2×10^−24^
Prostate cancer	29	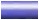	3.2×10^−21^
TNF signaling pathway	29	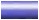	8.7×10^−19^
Bladder cancer	20	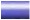	2.3×10^−18^
Chagas disease (American trypanosomiasis)	27	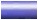	7.2×10^−17^
Toxoplasmosis	28	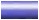	2.0×10^−16^
Nonsmall cell lung cancer	20	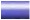	2.7×10^−15^
Hepatitis C	28	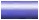	5.4×10^−15^

## Data Availability

The chemical ingredients of YCSND were extracted from TCMSP platform to support the findings of this study. The important nodes of YCSND compound-target network used to support the findings of this study are included within the article. The PPI network used to rank the importance of targets is performed by using STRING software. The degree of targets was collected from STRING software after setting the confidence level greater than 0.9 and rejecting the target protein independent of the network. The gene ontology (GO) functional enrichment analysis includes TOP10 of composition (CC), molecular function (MF), biological process (BP), and KEGG pathway enrichment used to elaborate the pharmacological mechanism of YCSD, which are included within the article. Besides, the rest of them used to support the findings of this study are included within the supplementary information file(s).

## Supplementary Materials

Supplementary Materials Supplementary 1. PPI combined score.

## References

[1] Chen S., Wu S., Li W. (2014). Investigation of the therapeutic effectiveness of active components in Sini decoction by a comprehensive GC/LC-MS based metabolomics and network pharmacology approaches. *Molecular BioSystems*.

[2] Wang X., Sun W., Sun H. (2008). Analysis of the constituents in the rat plasma after oral administration of Yin Chen Hao Tang by UPLC/Q-TOF-MS/MS. *Journal of Pharmaceutical and Biomedical Analysis*.

[3] Har C. W., Po J. (2003). Treatment of 22 Cases of Acute Hepatitis with Modified Yinchen Sini Decoction. *Journal of ZhenJiang Medical College*.

[4] Lee S., Chang B. (2013). Traditional chinese medicine network pharmacology: theory, methods and applications. *Chinese Journal of Natural Medicines*.

[5] Turner K. A., Manouchehri J. M., Kalafatis M. (2018). Sensitization of recombinant human tumor necrosis factor-related apoptosis-inducing ligand-resistant malignant melanomas by quercetin. *Melanoma Research*.

[6] Afifi N. A., Ibrahim M. A., Galal M. K. (2018). Hepatoprotective influence of quercetin and ellagic acid on thioacetamide-induced hepatotoxicity in rats. *Canadian Journal of Physiology and Pharmacology*.

[7] Lee Y., Beak S., Choi I., Sung J. (2018). Quercetin and its metabolites protect hepatocytes against ethanol-induced oxidative stress by activation of Nrf2 and AP-1. *Food Science and Biotechnology*.

[8] Altorki N. K., Subbaramaiah K., Dannenberg A. J. (2004). COX-2 inhibition in upper aerodigestive tract tumors. *Seminars in Oncology*.

[9] Fernández-Alvarez A., Llorente-Izquierdo C., Mayoral R. (2012). Evaluation of epigenetic modulation of cyclooxygenase-2 as a prognostic marker for hepatocellular carcinoma. *Oncogenesis*.

[10] Tolba R. H., Fet N., Yonezawa K. (2014). Role of preferential cyclooxygenase-2 inhibition by meloxicam in ischemia/reperfusion injury of the rat liver. *European Surgical Research*.

[11] Kuzumoto Y., Sho M., Ikeda N. (2005). Significance and therapeutic potential of prostaglandin E2 receptor in hepatic ischemia/reperfusion injury in mice. *Hepatology*.

[12] Motiño O., Francés D. E., Casanova N. (2018). Protective role of hepatocyte cyclooxygenase-2 expression against liver ischemia-reperfusion injury in mice. *Hepatology*.

[13] Gocher A. M., Azabdaftari G., Euscher L. M. (2017). Akt activation by Ca2+/calmodulin-dependent protein kinase kinase 2 (CaMKK2) in ovarian cancer cells. *The Journal of Biological Chemistry*.

[14] Mahmoud Y. I. (2018). Chronic cholestasis is associated with hypogonadism and premature ovarian failure in adult rats (cholestasis causes ovarian hypogonadism). *Ultrastructural Pathology*.

[15] Calderón-Montaño J. M., Burgos-Morón E., Pérez-Guerrero C., López-Lázaro M. (2011). A review on the dietary flavonoid kaempferol. *Mini-Reviews in Medicinal Chemistry*.

[16] Shoja M. H., Reddy N. D., Nayak P. G., Srinivasan K. K., Rao C. M. (2015). *Glycosmis pentaphylla* (Retz.) DC arrests cell cycle and induces apoptosis via caspase-3/7 activation in breast cancer cells. *Journal of Ethnopharmacology*.

[17] Guo G. M., Wong M. R. (2005). c-Jun and c-Fos and their association with human tumors. *International Journal of Genetics*.

[18] Juneja M., Ilm K., Schlag P. M., Stein U. (2013). Promoter identification and transcriptional regulation of the metastasis gene MACC1 in colorectal cancer. *Molecular Oncology*.

[19] Zhang H.-S., Yan B., Li X.-B. (2012). PAX2 protein induces expression of cyclin D1 through activating AP-1 protein and promotes proliferation of colon cancer cells. *The Journal of Biological Chemistry*.

[20] Rhee H., Kim H., Choi J. (2018). Keratin 19 expression in hepatocellular carcinoma is regulated by fibroblast-derived HGF via a MET-ERK1/2-AP1 and SP1 axis. *Cancer Research*.

[21] Huang T., Kang W., Zhang B. (2016). miR-508-3p concordantly silences NFKB1 and RELA to inactivate canonical NF-*Κ*B signaling in gastric carcinogenesis. *Molecular Cancer*.

[22] Huang X. P., Wu J. (2010). Study on the clinical significance of the expression of AP-1 and NF-*κ*B in hepatocellular carcinoma. *Chinese Journal of Cancer Prevention and Treatment*.

[23] Shen J., Zhang T., Cheng Z. (2018). Lycorine inhibits glioblastoma multiforme growth through EGFR suppression. *Journal of Experimental & Clinical Cancer Research*.

[24] Wong W. Q., Xu H. F., Lau Q. (2005). Relationship between epidermal growth factor and its receptors and liver cancer. *Journal of International Oncology*.

[25] Algül H., Tando Y., Schneider G., Weidenbach H., Adler G., Schmid R. M. (2002). Acute experimental pancreatitis and NF-*κ*B/Rel activation. *Pancreatology*.

[26] Zhang C., Guo X., Qin Y. (2010). Huangqi Injection reduces NF-*κ*B activity and down-regulates TNF-*α* mRNA expression in rats with acute pancreatitis. *World Chinese Journal of Digestology*.

[27] Vainer N., Dehlendorff C., Johansen J. S. (2018). Systematic literature review of IL-6 as a biomarker or treatment target in patients with gastric, bile duct, pancreatic and colorectal cancer. *Oncotarget *.

[28] Cheon Y. K., Cho Y. D., Moon J. H. (2007). Diagnostic utility of interleukin-6 (IL-6) for primary bile duct cancer and changes in serum IL-6 levels following photodynamic therapy. *American Journal of Gastroenterology*.

[29] McKenzie R. C., Venner T. J., Sauder D. N., Farkas-Himsley H. (1994). Augmentation of interleukin-6 (IL-6) expression in squamous carcinoma cells and normal human keratinocytes treated with recombinant anti-neoplastic protein (ACP). *Anticancer Reseach*.

[30] Reisi P., Eidelkhani N., Rafiee L., Kazemi M., Radahmadi M., Alaei H. (2017). Effects of doxepin on gene expressions of Bcl-2 family, TNF-*α*, MAP kinase 14, and Akt1 in the hippocampus of rats exposed to stress. *Research in Pharmaceutical Sciences*.

[31] Ren J., Yuan L., Wang Y., Chen G., Hu K. (2018). Benzyl sulforaphane is superior to sulforaphane in inhibiting the Akt/MAPK and activating the Nrf2/ARE signalling pathways in HepG2 cells. *Journal of Pharmacy and Pharmacology*.

[32] Harikrishnan H., Jantan I., Haque MA., Kumolosasi E. (2018). Anti-inflammatory effects of Phyllanthus amarus Schum. Thonn. through inhibition of NF-*κ*B, MAPK, and PI3K-Akt signaling pathways in LPS-induced human macrophages. *BMC Complementary and Alternative Medicine*.

[33] Sun Y.-W., Qiu H.-C., Ou M.-C., Chen R.-L., Liang G. (2018). Saponins isolated from Schizocapsa plantaginea inhibit human hepatocellular carcinoma cell growth in vivo and in vitro via mitogen-activated protein kinase signaling. *Chinese Journal of Natural Medicines*.

[34] Goyal A., Chauhan R. (2018). The dynamics of integration, viral suppression and cell-cell transmission in the development of occult Hepatitis B virus infection. *Journal of Theoretical Biology*.

[35] Ren J., Hu J., Cheng S. (2018). SIRT3 restricts hepatitis B virus transcription and replication through epigenetic regulation of covalently closed circular DNA involving suppressor of variegation 3-9 homolog 1 and SET domain containing 1A histone methyltransferases. *Hepatology*.

[36] Scotece M., Conde J., Abella V. (2018). Oleocanthal inhibits catabolic and inflammatory mediators in lps-activated human primary osteoarthritis (OA) chondrocytes through mapks/nf-*κ*b pathways. *Cellular Physiology and Biochemistry*.

[37] Ma J., Chen X., Xin G., Li X. (2019). Chronic exposure to the ionic liquid [C8mim]Br induces inflammation in silver carp spleen: Involvement of oxidative stress-mediated p38MAPK/NF-*κ*B signalling and microRNAs. *Fish & Shellfish Immunology*.

[38] Jiang L., Zhu J. S. (2002). New research progress on correlation between prognosis of acute pancreatitis and cellular immune factors. *World Chinese Journal of Digestology*.

